# Early pregnancy loss in Belagavi, Karnataka, India 2014–2017: a prospective population-based observational study in a low-resource setting

**DOI:** 10.1186/s12978-018-0525-4

**Published:** 2018-06-22

**Authors:** Sangappa M. Dhaded, Manjunath S. Somannavar, Jane P. Jacob, Elizabeth M. McClure, Sunil S. Vernekar, S. Yogesh Kumar, Avinash Kavi, Umesh Y. Ramadurg, Janet L. Moore, Dennis P. Wallace, Richard J. Derman, Robert L. Goldenberg, Shivaprasad S. Goudar

**Affiliations:** 1 Women’s and Children’s Health Research Unit, J N Medical College, KLE Academy of Higher Education and Research, Belagavi, Karnataka India; 2 Thomas Jefferson University, Philadelphia, PA USA; 3 RTI International, Durham, NC USA; 4 Columbia University, New York, NY USA

**Keywords:** Miscarriage, Medically terminated pregnancy, Early pregnancy loss, India

## Abstract

**Background:**

The prevalence of early pregnancy loss through miscarriage and medically terminated pregnancy (MTP) is largely unknown due to lack of early registration of pregnancies in most regions, and especially in low- and middle-income countries. Understanding the rates of early pregnancy loss as well as the characteristics of pregnant women who experience miscarriage or MTP can assist in better planning of reproductive health needs of women.

**Methods:**

A prospective, population-based study was conducted in Belagavi District, south India. Using an active surveillance system of women of childbearing age, all women were enrolled as soon as possible during pregnancy. We evaluated rates and risk factors of miscarriage and MTP between 6 and 20 weeks gestation as well as rates of stillbirth and neonatal death. A hypothetical cohort of 1000 women pregnant at 6 weeks was created to demonstrate the impact of miscarriage and MTP on pregnancy outcome.

**Results:**

A total of 30,166 women enrolled from 2014 to 2017 were included in this analysis. The rate of miscarriage per 1000 ongoing pregnancies between 6 and 8 weeks was 115.3, between 8 and 12 weeks the miscarriage rate was 101.9 per 1000 ongoing pregnancies and between 12 and 20 weeks the miscarriage rate was 60.3 per 1000 ongoing pregnancies. For those periods, the MTP rate was 40.2, 45.4, and 48.3 per 1000 ongoing pregnancies respectively. The stillbirth rate was 26/1000 and the neonatal mortality rate was 24/1000. The majority of miscarriages (96.6%) were unattended and occurred at home. The majority of MTPs occurred in a hospital and with a physician in attendance (69.6%), while 20.7% of MTPs occurred outside a health facility. Women who experienced a miscarriage were older and had a higher level of education but were less likely to be anemic than those with an ongoing pregnancy at 20 weeks. Women with MTP were older, had a higher level of education, higher parity, and higher BMI, compared to those with an ongoing pregnancy, but these results were not consistent across gestational age periods.

**Conclusions:**

Of women with an ongoing pregnancy at 6 weeks, about 60% will have a living infant at 28 days of age. Two thirds of the losses will be spontaneous miscarriages and one third will be secondary to a MTP. High maternal age and education were the risk factors associated with miscarriage and MTP.

**Trial registration:**

The trial is registered at clinicaltrials.gov. ClinicalTrial.gov Trial Registration: NCT01073475.

## Background

Miscarriage is a public health issue throughout the world. Miscarriages, often defined as spontaneous pregnancy losses before 20 weeks gestational age, are estimated to occur in almost 1 in 3 pregnancies, many of which occur before the pregnancy is clinically recognized [1, 2]. This may be especially true in developing countries where women do not have ready access to health care providers.

Various factors have been associated with miscarriage, including the number of previous miscarriages, nutritional status, and the level of personal and community support [3]. Miscarriages may also be caused by various environmental, anatomical, and genetic factors. Exposure to certain infections can also increase the chance of a miscarriage [4]. Additionally, many studies have shown advanced maternal age to be significantly related to risk of miscarriage [3].

High rates of malnutrition and poor-nutrition, often measured by body mass index and hemoglobin levels among women in developing countries, may be contributing factors to high rates of early pregnancy loss [5]. Additionally, lack of health resources, including antenatal care, might also contribute to increased rates of miscarriage.

India, in particular, has reported high rates of miscarriage, particularly for women who have had previous miscarriages or unsafe abortions [6]. A household survey conducted in Bihar, India found that miscarriage rates were reported to be 46 per 1000 pregnancies (4.6%), but registration tended to occur later in pregnancy [6]. In contrast, an epidemiological study based in major cities throughout India found the prevalence of recurrent spontaneous miscarriages among Indian women to be as high as 32% [4]. None of these studies mentioned the gestational age at enrolment.

Another form of pregnancy loss is medically terminated pregnancy (MTP), which can be performed either in a hospital or at home, through medication or surgery. In India, MTPs can legally occur until 20 weeks of gestational age [7]. A recent study looked at abortion rates in health facilities within six states in India and found that MTPs accounted for 33% of pregnancies [8].

Thus, statistics on miscarriages and MTPs in India vary widely across the literature, partially due to difficulty in obtaining reliable early gestational age data among vulnerable, rural populations. Many of these studies include data from developed regions of India. Therefore, we do not have an accurate understanding of pregnancy outcomes in India’s rural settings. Additionally, while studies on specific clinical contributors, such as nutrition and previous miscarriages exist, few population-based studies are available examining the rates of miscarriage and MTP and their association with maternal characteristics. Better understanding the impact of medical services on the pregnancies of rural woman, as well as the interplay of societal structure and personal health in the context of rural, developing regions, can help better target the complexities of miscarriages and MTP in these settings. These issues affect a large proportion of women throughout rural India, as well women in other parts of the world, and merit a nuanced and multidimensional approach.

Our study aims to identify the prevalence and characteristics of women who experience miscarriage or MTP in rural settings within Belagavi, India.

## Methods

This study was conducted as part of the Global Network’s Maternal Newborn Health Registry (MNHR), a population based, observational study conducted in six low-resource countries, including India [9]. The objective of the MNHR is to enrol all pregnant women residing within defined geographic areas, study clusters, which generally have 300 to 500 deliveries per year. This analysis includes data collected from pregnant women enrolled in the Belagavi MNHR clusters from 2014 to 2017.

All pregnant women residing within a study cluster, or giving birth within the cluster, were approached as early as possible during their pregnancy for inclusion in the MNHR. Following informed consent, women were followed by study staff, known as registry administrators (RAs). The RAs enrolled consenting pregnant women and completed perinatal outcome forms for each woman enrolled in the MNHR. RAs collected information on prenatal services and the health status of the mother, including age, hemoglobin level, weight, height, and previous pregnancies. Pregnancy outcomes and mode of delivery were also recorded.

The RAs attempted to register the women as early in the pregnancy as possible, using an ongoing registry of women likely to get pregnant and frequent pregnancy testing among those likely to get pregnant [10]. The data were then reviewed and cleaned by research staff and then entered in a local secure study computer where additional edits were performed. Data were then transmitted to a central data-coordinating center, RTI International, where additional edits were performed and resolved by the site.

### Statistical analyses

We calculated the body mass index (BMI) from the mother’s weight and height in kg/m^2^ based on measurements generally taken < 12 weeks gestation. Hemoglobin measurements were taken at the first ANC visit and categorized. Miscarriages were defined as any spontaneous loss prior to 20 weeks. A medical termination included the elective termination of pregnancy whether the termination was performed medically or surgically. A stillbirth was defined as the birth of a baby at 20 weeks or more with no signs of life. The gestational age was based on ultrasound, if available, or otherwise the date of the last menstrual period. Women with a missing gestational age or enrolled at delivery were excluded.

Because women enrol in care at various times in pregnancy, and it appears that those who have an early miscarriage or MTP may not enrol at all, a modelling exercise to account for women not entering care was needed. To estimate the outcome of 1000 hypothetical pregnancies ongoing at 6 weeks, we applied the rates of miscarriage and MTP found in the ongoing pregnancies at 6 weeks to 7 weeks 6 days to the entire population of 1000 pregnancies. To estimate the outcomes of the remaining hypothetical pregnancies ongoing at 8 weeks, we applied the rates of miscarriage and MTP found in the ongoing pregnancies at 8 weeks to 11 weeks 6 days to the remaining ongoing pregnancies. To estimate the outcome of the remaining hypothetical pregnancies ongoing at 12 weeks we applied the rates of miscarriage and MTP found in the ongoing pregnancies at 12 weeks to 19 weeks 6 days to the remaining ongoing pregnancies. We also applied the rates of stillbirth and neonatal mortality to the remaining pregnancies at 20 weeks to determine the number of living infants at 28 days of age.

We were also interested in the characteristics of woman having miscarriages and MTPs. Descriptive analyses included frequency and distribution of risk factors for women having miscarriages and MTPs within each gestational age category. We analysed the individual risk relationship of each variable with the risk of miscarriage and MTP for all pregnancies terminating from 6 weeks to 19 weeks 6 days within the gestational age groups of 6 weeks to 7 weeks 6 days, 8 weeks to 11 weeks 6 days and 12 weeks to 19 weeks 6 days. For the outcome variables of miscarriage and MTP, relative risks (RR) and 95% confidence intervals (CI) were obtained from generalized linear models with a binomial distribution assumption and log link for binary outcomes accounting for a single risk factor and controlling for each of the variables as well as cluster as a random effect. All analyses were performed with SAS (Cary, NC).

## Results

**Fig. 1:**
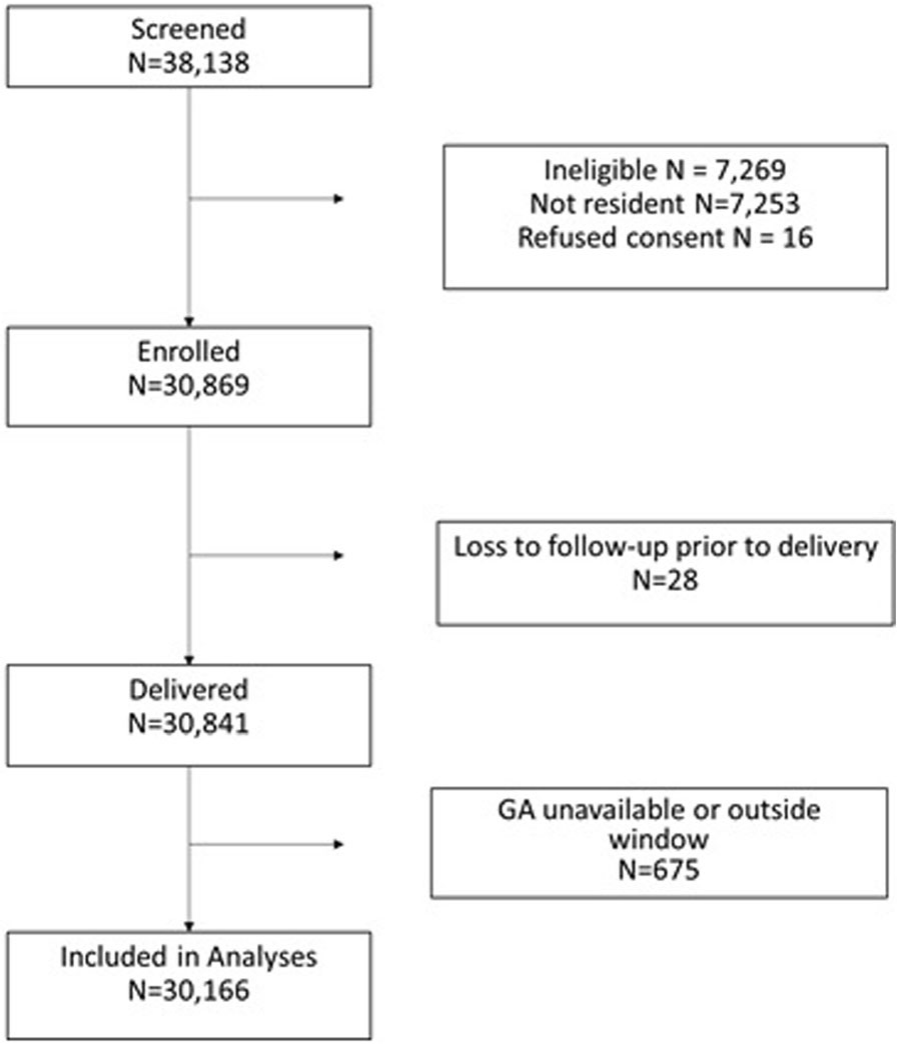
Flow diagram of pregnant women enrolled in the Belagavi MNHR Study, 2014–2017

A total of 38,138 women were screened and of those, 30,869 (81%) were eligible and consented. Most of those who were ineligible were not residents of the catchment area (Fig. 1). Of those, 30,166 (97.8%) women had gestational age information available and were included in the analyses. Of these women, 9.2% were enrolled prior to 6 weeks gestation, 28.9% between 6 weeks and 7 weeks 6 days, 36.4% between 8 weeks and 11 weeks 6 days, 19.9% between 12 weeks and 19 weeks 6 days, and 5.6% at or after 20 weeks.

Table 1 summarizes the antenatal care (ANC) and obstetric characteristics for women with miscarriages and MTPs prior to 20 weeks and ongoing pregnancies at 20 weeks. Among women with a miscarriage, 93.9% had at least one ANC visit, while 95.2 and 100% of women with MTPs and ongoing pregnancies at 20 weeks received at least one ANC visit. Among women with a MTP, 84.8% were performed by a physician, but 13.2% occurred without a physician or nurse present. For women with pregnancies ongoing at 20 weeks, 61.1% were ultimately delivered by a physician and 2.7% were unattended. Among women having a miscarriage, 2.8% occurred within a hospital, 0.9% at a health center and 96.3% at home. For those with a MTP, 69.9% occurred at a hospital, 9.7% at a health center and 20.7% at home. Finally, among those with an ongoing pregnancy at 20 weeks, the delivery ultimately occurred in a hospital for 71.3%, at health center for 24.3 and 4.4% occurred at home.

**Table 1:** Antenatal and obstetric care for women with a miscarriage or MTP vs those with an ongoing pregnancy at 20 weeks gestation

	Miscarriage	MTP	Ongoing pregnancy at 20 weeks GA
Enrolled, *N*	3598	1894	24,674
At least one ANC visit (%)	93.9	95.2	100.0
ANC visits (%)			
0	6.1	4.8	0.0
1–2	93.0	91.4	5.2
> 2	0.9	3.8	94.8
Delivery attendant (%)			
Physician	2.6	84.8	61.1
Nurse/Nurse midwife	0.7	1.2	35.8
TBA	0.1	0.7	0.4
Family/Self delivery/Other	96.6	13.2	2.7
Delivery location, *N* (%)			
Hospital	2.8	69.6	71.3
Clinic/Health center	0.9	9.7	24.3
Home/Other	96.3	20.7	4.4

*MTP* medically terminated pregnancy

*ANC* antenatal care

*GA* gestational age

*TBA* traditional birth attendant

We next estimated the rates of miscarriage and MTP per 1000 ongoing pregnancies by gestational age categories (Table 2). Among women who were enrolled by 6 weeks gestation, the rate of miscarriage between 6 weeks and 7 weeks 6 days was 115.3/1000, and the MTP rate was 45.4/1000. Among those with an ongoing pregnancy at 8 weeks, the rate of miscarriage between 8 and 11 weeks 6 days was 101.9/1000, and the rate of MTP was 48.3/1000. Finally, among those with an ongoing pregnancy at 12 weeks, the rate of miscarriage between 12 weeks and 19 weeks 6 days was 60.3/1000 and the MTP rate was 40.2/1000.

**Table 2:** Miscarriage and MTP rates per 1000 ongoing pregnancies among women with an ongoing pregnancy at 6, 8 and 12 weeks gestation

	Number (n/N)	Rate per 1000 ongoing pregnancies
Enrolled, N	30,166	
Enrolled by 6 weeks without loss < 6 weeks, N	2775	
Miscarriage 6,0–7,6 weeks	307/2663	115.3
MTP 6,0–7,6 weeks	112/2468	45.4
Enrolled by 8 weeks without loss < 8 weeks, N	10,452	
Miscarriage 8,0–11,6 weeks	1019/9996	101.9
MTP 8,0–11,6 weeks	456/9433	48.3
Enrolled by 12 weeks without loss < 12 weeks, N	19,200	
Miscarriage 12,0–19,6 weeks	1114/18,473	60.3
MTP 12,0–19,6 weeks	727/18,086	40.2

*MTP* medically terminated pregnancy

For this analysis, the denominators for miscarriage and MTP are different because in each time-period, the number of miscarriages were excluded from the denominator for MTPs, and for MTPs, the number of miscarriages were excluded from the denominator

**Fig. 2:**
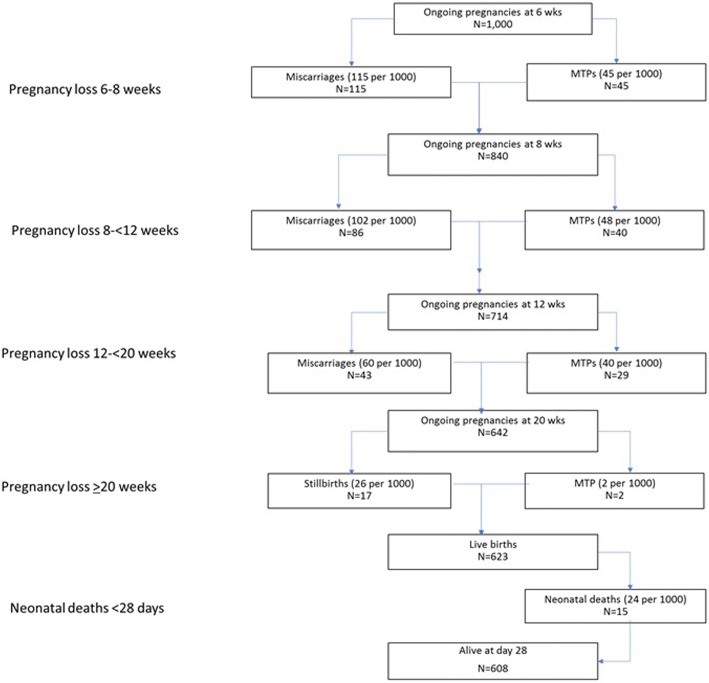
Miscarriages and medical terminations of pregnancy as well as stillbirths and neonatal deaths for a hypothetical cohort of 1000 ongoing pregnancies at 6 weeks gestation based on the rates of early pregnancy loss in Belagavi, India

Applying these rates to a hypothetical population of 1000 ongoing pregnancies at 6 weeks gestation (Fig. 2), we calculated that between 6 weeks to 7 weeks 6 days, there would be 115 miscarriages and 45 MTPs leaving 840 ongoing pregnancies at 8 weeks. With 840 ongoing pregnancies at 8 weeks gestation, there would then be an additional 86 miscarriages and 40 MTPs between 8 and 11 weeks 6 days gestation, leaving 714 ongoing pregnancies at 12 weeks gestation. Between 12 weeks to 19 weeks 6 days, there would be 43 miscarriages and 29 abortions leaving 642 ongoing pregnancies at 20 weeks gestation. In this population, an additional 2 MTPs were reported after 20 weeks gestation, leaving 640 pregnancies. During the study, the stillbirth rate was estimated to be 26 per 1000 and the 28-day neonatal mortality rate was 24 per 1000 (data not shown). Applying these rates to our hypothetical population, after the additional 17 stillbirths and 15 neonatal deaths were substracted from the ongoing pregnancies at 20 weeks, 608 infants would be alive at 28 days of age.

We next evaluated characteristics associated with miscarriage for three categories of gestational age (6 weeks to 7 weeks 6 days, 8 weeks to 11 weeks 6 days and 12 weeks to 19 weeks 6 days). In this analysis, the characteristics of women with a miscarriage within the gestational age category were compared to those with an ongoing pregnancy. The adjusted RR and 95% CI for each group are presented (Table 3). Among women > 30 years of age, there was an increasing risk of miscarriage as the gestational age of the pregnancy increased from 6 weeks to 7 weeks 6 days, to 8 weeks to 11 weeks 6 days, and to 12 weeks to 19 weeks 6 days. Women with a secondary education appeared more likely to have a miscarriage at 12 to 19 weeks 6 days, and women with a BMI > 25 kg/m^2^ appeared less likely to have a miscarriage at 6 weeks to 7 weeks 6 days. Women with hemoglobin levels < 9 g/dl were less likely to have a miscarriage at 6 weeks to 7 weeks 6 days. Those with a hemoglobin level from 9 to 11 g/dl were less likely to have a miscarriage at 6 weeks to 7 weeks 6 days and at 8 weeks to 11 weeks 6 days. Those with a hemoglobin < 9 g/dl had a lower risk of miscarriage in all gestational age groups although only in the earliest gestational age group were the results significant. Otherwise, there were no significant differences by the gestational age of the miscarriage observed.

**Table 3:** Relative risk (RR) of miscarriage vs ongoing pregnancy in three gestational age groups by women’s characteristics

	Miscarriage v. Ongoing 6,0–7,6 Weeks RR (95% CI)	Miscarriage v. Ongoing 8,0–11,6 Weeks RR (95% CI)	Miscarriage v. Ongoing 12,0–19,6 Weeks RR (95% CI)
Maternal age (years)			
≤ 20	1.26 (0.94, 1.69)	1.12 (0.95, 1.33)	0.94 (0.80, 1.10)
21–25	REF	REF	REF
26–30	1.11 (0.80, 1.53)	1.06 (0.90, 1.26)	1.09 (0.93, 1.29)
> 30	1.17 (0.64, 2.14)	1.42 (1.05, 1.93)	1.81 (1.37, 2.38)
Maternal education			
No formal education	0.93 (0.59, 1.46)	0.94 (0.71, 1.23)	1.12 (0.86, 1.46)
Primary	REF	REF	REF
Secondary	0.95 (0.69, 1.32)	1.10 (0.92, 1.31)	1.42 (1.20, 1.68)
University+	1.00 (0.61, 1.62)	0.89 (0.68, 1.16)	0.93 (0.68, 1.27)
Parity			
0	0.78 (0.57, 1.07)	0.73 (0.60, 0.89)	0.89 (0.75, 1.06)
1–2	REF	REF	REF
> 2	1.30 (0.88, 1.91)	1.11 (0.84, 1.48)	1.08 (0.83, 1.41)
BMI (kg/m^2)^			
Underweight, < 18.5 kg/m^2^	1.00 (0.85, 1.19)	1.02 (0.96, 1.08)	1.00 (0.86, 1.17)
Normal weight, 18.5–25 kg/m^2^	REF	REF	REF
Overweight /obese, > 25 kg/m^2^	0.61 (0.41, 0.91)	0.89 (0.68, 1.16)	0.99 (0.77, 1.29)
Hemoglobin (gm/dl)			
Severe/Moderate, ≤ 9 g/dl	0.77 (0.61, 0.98)	0.86 (0.70, 1.06)	0.93 (0.74, 1.17)
Mild, > 9–11 g/dl	0.75 (0.61, 0.92)	0.81 (0.70, 0.93)	1.00 (0.86, 1.15)
Normal, > 11 g/dl	REF	REF	REF

*BMI* body mass index

*REF* reference group

Among women with an MTP, ages 26–30 and > 30 were generally associated with an increased rate of MTP, as was having a secondary or university education compared to those with a primary education. Nulliparity was associated with decreased risk of MTP in the two lowest gestational age groups. Parity > 2 was associated with a higher risk of MTP but was significant only at 12 to 19 weeks 6 days. Having a BMI <  18.5 kg/m^2^ was associated with a lower rate of MTP at 6 weeks to 7 weeks 6 days and nearly so at 8 weeks to 11 weeks 6 days. Otherwise, there were no significant differences for MTP by gestational age (Table 4).

**Table 4:** Relative risk (RR) of medically terminated pregnancy (MTP) vs ongoing pregnancy in 3 gestational age groups by women’s characteristics

	MTP v. Ongoing 6,0–7,6 Weeks RR (95% CI)	MTP v. Ongoing 8,0–11,6 Weeks RR (95% CI)	MTP v. Ongoing 12,0–19,6 Weeks RR (95% CI)
Maternal age (years)			
≤ 20	1.24 (0.65, 2.36)	1.12 (0.78, 1.61)	0.85 (0.67, 1.08)
21–25	REF	REF	REF
26–30	2.02 (1.08, 3.77)	1.22 (0.99, 1.52)	1.15 (1.00, 1.32)
> 30	2.76 (1.34, 5.68)	2.07 (1.41, 3.05)	2.19 (1.76, 2.74)
Maternal education			
No formal education	1.02 (0.31, 3.36)	1.02 (0.59, 1.77)	1.00 (0.71, 1.41)
Primary	REF	REF	REF
Secondary	1.32 (0.54, 3.26)	1.63 (1.16, 2.30)	1.35 (1.04, 1.75)
University+	1.27 (0.48, 3.41)	1.82 (1.01, 3.28)	1.52 (1.05, 2.21)
Parity			
0	0.19 (0.08, 0.45)	0.52 (0.37, 0.74)	0.98 (0.81, 1.17)
1–2	REF	REF	REF
> 2	1.22 (0.65, 2.31)	1.42 (0.97, 2.06)	1.28 (1.00, 1.63)
BMI (kg/m^2^)			
Underweight, < 18.5 kg/m^2^	0.65 (0.44, 0.96)	0.79 (0.63, 1.01)	1.02 (0.88, 1.18)
Normal weight, 18.5–25 kg/m^2^	REF	REF	REF
Overweight and obese, > 25 kg/m^2^	1.06 (0.51, 2.20)	0.81 (0.57, 1.16)	1.15 (0.95, 1.39)
Hemoglobin (gm/dl)			
Severe/Moderate, ≤ 9 g/dl	1.57 (0.87, 2.83)	0.75 (0.46, 1.24)	1.04 (0.91, 1.19)
Mild, > 9–11 g/dl	1.16 (0.76, 1.78)	0.79 (0.61, 1.01)	0.93 (0.82, 1.05)
Normal, > 11 g/dl	REF	REF	REF

*BMI* body mass index

*REF* reference group

*MTP* medically terminated pregnancy

## Discussion

In this study, we demonstrated that of 1000 ongoing pregnancies at 6 weeks gestational age in Belagavi, India, there will only be about 600 living infants at the end of the perinatal period. Spontaneous miscarriages account for about 2/3 of the losses and MTP for about 1/3. Stillbirths and neonatal deaths account for much smaller proportions of the losses. Miscarriage and MTP were more common at 6 to 8 weeks gestational age compared to later time-periods.

We found that a number of factors were associated with miscarriage. Older women and women with a secondary education were often at an increased risk for miscarriage when compared to women who had ongoing pregnancies. Women with low parity were often at decreased risk. BMI > 25 kg/m^2^ was associated with a lower risk of miscarriage at 6 weeks to 7 weeks 6 days, but not at other times. For unexplained reasons, lower hemoglobin levels were associated with a lower risk of miscarriage prior to 12 weeks.

Our results also showed that older women, those with higher education, those with higher parity were more likely to have a MTP. Women with a BMI <  18.5 kg/m^2^ were less likely to have an MTP at 6 weeks to 7 weeks 6 days. Hemoglobin levels were not associated with rates of MTP in any time-period.

Our results are broadly comparable to those in other studies. A study in a low-income country which looked at the rate of miscarriages by gestational age week, had a mean age of enrolment of nearly 14 weeks, while in our study, nearly 80% of the subjects were enrolled prior to 12 weeks [11]. Many studies estimate that around 30% of pregnancies end in a miscarriage, while in our study about 28% of ongoing pregnancies at 6 weeks were estimated to end in a miscarriage. However, the gestational age of women’s enrolment and the gestational age when miscarriages become stillbirths or live births in various studies make more exact comparisons impossible. The risks for miscarriage found in our study were similar to those previously reported and included older age and higher parity. The results in our study were often not consistent across the gestational periods studied while this information was not available in most other low-income country studies [3, 4].

### Strengths and limitations

Because of ongoing surveillance systems of women likely to become pregnant and the frequent pregnancy testing, we believe we have captured ongoing pregnancies as early in pregnancy as possible in a low-income country study. The rates of miscarriage and MTP likely represent the actual rates of miscarriage and MTP in ongoing pregnancies in each of the time-periods studied. Nevertheless, because pregnant women present for care at various times in pregnancy, it was necessary to use available data to construct a hypothetical cohort of 1000 women with an ongoing pregnancy at 6 weeks to enable us to calculate the overall losses expected. Therefore, one of the potential weaknesses of the study is that the rates of miscarriage and MTP in this model are estimates. However, these estimates are based on accurate population-based data of pregnancies enrolled quite early. In general, we use self-reported data and have not validated whether or when a miscarriage or MTP actually occurred. However, because we followed the women closely, we believe these data are reasonably accurate. In some of the time-periods and with certain of the characteristics, the numbers in the cells are small and the resultant confidence intervals are relatively large, thus precision may be limited. Since the gestational age data were often obtained by LMP without ultrasound confirmation, the number of women in each gestational age group may represent an approximation.

## Conclusion

Of 1000 ongoing pregnancies at 6 weeks gestation, nearly 40% will be lost prior to 20 weeks gestation, two thirds to miscarriage and one third to MTP. A much smaller percentage will be lost later to stillbirth and neonatal mortality. Risk factors for miscarriage include higher maternal age and education. In this study, for reasons not obvious to us, lower haemoglobin levels appear to be related to lower rates of miscarriage. Higher maternal age, parity, and BMI were associated with MTP. These results should enable health systems to evaluate the resources necessary to provide appropriate care for all pregnant women including those having spontaneous miscarriages and undergoing MTP.

## Abbreviations

ANC: Antenatal Care
BMI: Body Mass Index
MNHR: Maternal Newborn Health Registry
MTP: Medically Terminated Pregnancy
